# Cancer incidence and mortality in 23 000 patients with type 1 diabetes in the UK: Long‐term follow‐up

**DOI:** 10.1002/ijc.34548

**Published:** 2023-05-15

**Authors:** Anthony J. Swerdlow, Michael E. Jones, Stefan D. Slater, Andrew C. F. Burden, Johannes L. Botha, Norman R. Waugh, Andrew D. Morris, Wendy Gatling, Kathleen M. Gillespie, Christopher C. Patterson, Minouk J. Schoemaker

**Affiliations:** ^1^ Division of Genetics and Epidemiology The Institute of Cancer Research London UK; ^2^ Division of Breast Cancer Research The Institute of Cancer Research London UK; ^3^ 80 Whitehouse Rd Edinburgh UK; ^4^ Top Orchard, Broadhembury Honiton Devon UK; ^5^ Meanwood Leeds UK; ^6^ Division of Health Sciences University of Warwick Coventry UK; ^7^ Health Data Research UK London UK; ^8^ Department of Diabetes Poole Hospital NHS Trust Dorset UK; ^9^ Diabetes and Metabolism Bristol Medical School, University of Bristol Bristol UK; ^10^ Department of Epidemiology and Public Health Queen's University Belfast UK; ^11^ Global Database Studies, Real World Solutions at IQVIA Netherlands

**Keywords:** cancer, cohort, type 1 diabetes

## Abstract

Type 2 diabetes is associated with raised risk of several cancers, but for type 1 diabetes risk data are fewer and inconsistent We assembled a cohort of 23 473 UK patients with insulin‐treated diabetes diagnosed at ages <30, almost all of whom will have had type 1 diabetes, and for comparison 5058 diagnosed at ages 30 to 49, of whom we estimate two‐thirds will have had type 2, and followed them for an average of 30 years for cancer incidence and mortality compared with general population rates. Patients aged <30 at diabetes diagnosis had significantly raised risks only for ovarian (standardised incidence ratio = 1.58; 95% confidence interval 1.16‐2.11; *P* < .01) and vulval (3.55; 1.94‐5.96; *P* < .001) cancers, with greatest risk when diabetes was diagnosed at ages 10‐14. Risks of cancer overall (0.89; 0.84‐0.95; *P* < .001) and sites including lung and larynx were significantly diminished. Patients diagnosed with diabetes at ages 30 to 49 had significantly raised risks of liver (1.76;1.08‐2.72) and kidney (1.46;1.03‐2.00) cancers, and reduced risk of cancer overall (0.89; 0.84‐0.95). The raised ovarian and vulval cancer risks in patients with type 1 diabetes, especially with diabetes diagnosed around pubertal ages, suggest possible susceptibility of these organs at puberty to metabolic disruption at diabetes onset. Reduced risk of cancer overall, particularly smoking and alcohol‐related sites, might reflect adoption of a healthy lifestyle.

## Graphical abstract

What's new?

A cohort of 23 000 UK patients with insulin‐treated diabetes diagnosed 1972 to 1993 at ages <30, therefore almost all with type 1 diabetes, were followed for average 30 years, considerably longer than in any published study. Unlike other studies, analyses were possible on age at diabetes diagnosis. Risks were significantly raised for incidence of ovarian and vulval cancers, especially if diabetes was diagnosed at ages 10 to 14, that is, likely around puberty, but were significantly decreased for cancer overall.

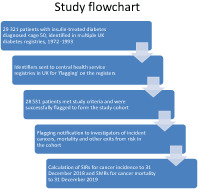

**Abbreviations**
BDA: British Diabetic Association
BSO: Business Services Organisation
ICD: international classification of diseases
NHSCR: national health service central registers
SIR: standardised incidence ratio
SMR: standardised mortality ratio
UK: United Kingdom

## 1 INTRODUCTION

Diabetes mellitus is one of the major chronic diseases of Western and increasingly of other populations. It is, however, the consequence of two very different disease processes, type 1 (insulin‐dependent) and type 2 (non‐insulin‐dependent), (and several much rarer types), with different metabolic and hormonal characteristics and treatments that might affect cancer risk. Most studies of cancer in patients with diabetes have concerned type 2 diabetes, far the more common type, or have been of patients with diabetes overall and therefore effectively of type 2.^1^, ^2^, ^3^, ^4^ These have shown raised risks of several tumours including, liver, pancreatic, colorectal, renal and endometrial cancers,^1^, ^2^, ^3^, ^4^, ^5^ but could reflect confounding by alcohol consumption and obesity, which are causes of this type of diabetes. There has been far less research on cancer incidence^5^, ^6^, ^7^, ^8^, ^9^, ^10^, ^11^, ^12^ and mortality^5^, ^13^ in patients with type 1 diabetes, and these have been difficult to interpret because of uncertainty about the proportion of subjects who were truly type 1. Proxy measures have been used to categorise diabetes type, for instance by including as type 1 all patients with diabetes incident under age 40 or 50 irrespective of treatment type.^8^, ^12^ However, most patients with diabetes incident at ages 30 and above are actually type 2,^14^, ^15^, ^16^ and hence cancer risks in several published cohorts may substantially reflect contamination by type 2 subjects.

Few data have been published on cancer risks by duration since diagnosis of type 1 diabetes,^9^, ^10^, ^12^ and these have been problematic both because of the diagnostic misclassification described above, and because hospitalisation or unspecified events have been used to categorise when diabetes was diagnosed, rather than using actual date of diagnosis. Duration is important because most known cancer causes act to raise risk over decades, not in the early years after first exposure, and this is the more so for exposures starting in childhood, when type 1 diabetes incidence is greatest. Also, in the immediate period after diabetes diagnosis, there is potential for reverse causation or detection bias, which could be uncovered by duration analyses.

We have therefore analysed cancer incidence and mortality risks in the Diabetes UK (formerly British Diabetic Association [BDA]) cohort, which includes over 20 000 patients with type 1 diabetes for whom date of type 1 diagnosis was recorded, and hence duration of diabetes known. The original cohort also included over 5000 insulin‐treated patients diagnosed at ages 30 to 49, and we analysed follow‐up of these too, to provide a comparison, albeit not as large, of mainly type 2 patients from the same sources. Cancer follow‐up in the cohort was last published 17 years ago.^17^ The follow‐up now includes over three times as many cancers and over twice as many cancer deaths as in that publication.

## 2 MATERIALS AND METHODS

The study cohort was formed by combining registrations from several registers of UK‐resident patients with insulin‐treated diabetes identified during 1972 to 1993. The largest was a register of 12 891 children compiled by the BDA during 1972 to 1986. The remainder were population‐based geographical registers from several parts of the UK.^18^ We combined these registers, for patients with diabetes diagnosed at ages under 50, removed overlaps, and, with appropriate ethics committee approval, sent identification details of the subjects to the National Health Service Central Registers (NHSCRs) for England and Wales, and Scotland, and the Central Services Agency, now the Business Services Organisation (BSO), for Northern Ireland. The NHSCRs and BSO hold virtually complete population registers for their countries, and hence provided us with “flagging” information on deaths and emigrations in the cohort since study entry and (except in Northern Ireland) on incident cancers since 1971. Sites of cancers and underlying causes of death were coded according to the International Classification of Diseases (ICD) revision appropriate to the date of the event, and we then bridge‐coded them to ICD 9.^19^ For each cohort member, we calculated person‐years at risk by 5‐year age group, sex, calendar year and country of residence, beginning at the date of registration in the study or age 1 year, whichever was later, and ending at December 31, 2019 or the date of 85th birthday, death, emigration or other loss to follow‐up, if earlier. Follow‐up was censored at age 85 because information on cause of death is relatively unreliable beyond that age. We omitted person‐years and deaths under 1 year of age because national mortality rates were not available by subdivisions of this age group, and underlying cause was not coded for neonatal deaths in England and Wales during most of the study period.

We then assessed cancer mortality risks by calculating standardised mortality ratios (SMRs) as the ratio of the number of deaths observed to the number expected from application of age, sex, calendar year and country specific person‐years at risk in the cohort to the corresponding mortality rates in the general population of England and Wales (for the English and Welsh cases) or Scotland (for the Scottish and Northern Irish cases) (since computerised death rates were not available for Northern Ireland). Standardised incidence ratios (SIRs) were calculated similarly, except that follow‐up was censored at 31 December 2018, because more recent cancer registrations were not yet complete, and Northern Ireland was omitted because cancer incidence flagging was not available there.

For initial assessment of cancer risks we analysed separately subjects with diabetes onset at ages under 30, and aged 30 to 49, because at the period of diabetes incidence in this cohort, this approximately divided those who would have been almost entirely type 1 diabetes from those who would have been largely type 2.^20^ We also assessed cancer risks by duration since diagnosis and by 10‐year age group of diabetes diagnosis, and for females we analysed risk separately for ages 10‐14 and 15‐19 at diagnosis, because in the UK menarche occurs at ages 10‐14 in about 90% of girls.^21^ Additionally, following the observation by Leete et al^22^ that patients with diabetes incident at ages under 30 can be subdivided into three immunologically separate groups based on age at diagnosis, 0‐6, 7‐12 and 13‐29, we analysed these three age‐groups separately. To examine whether the cancer diagnosis might have been made as a consequence of the clinical work‐up after diabetes was diagnosed (or vice versa), we conducted analyses of cancer incidence risks in the first year after diabetes diagnosis.

We used the above standard statistical methods for cohort analysis, without smoothing or “correction” of SIRs, and used standard quinquennial and decennial cut‐points (except where others have reported specific immunologically relevant cut‐points,^22^ or we were examining for diagnostic artefact at the time of diagnosis), to allow comparison with the literature and allow potential meta‐analysis, and to avoid data‐driven sub‐analyses. We calculated 95% confidence intervals and tested for trend assuming a Poisson distribution.^23^ All *P* values presented are 2‐sided. All statistical analyses were performed using Stata IC Version 16.0.^24^

## 3 RESULTS

There were 29 321 patients on the BDA cohort register aged under 50 years at diagnosis, of whom we excluded 29 because their diabetes was secondary to another disease such as cystic fibrosis, 211 because of inadequate data at registration, and 550 because their records could not be traced at the NHSCRs or BSO. This left 28 531 patients who formed the study cohort. Most (20444) had been diagnosed at ages under 20 years; 22 648 had been diagnosed during 1970 to 1989 and 2414 later than this (Table 1). Slightly more were male (15476) than female (13055).

**TABLE 1:** Cohort^a^ by age, sex, year of diagnosis of diabetes and other descriptive variables

Characteristics		Age at diabetes diagnosis (years)			
		0‐29		30‐49	
		Males	Females	Males	Females
Age at diagnosis of diabetes (years)	0‐9	5132	4796		
	10‐19	5667	4849		
	20‐29	1739	1290		
	30‐39			1594	1051
	40‐49			1344	1069
Year of diagnosis of diabetes	<1960	502	421	176	153
	1960‐1969	699	615	499	404
	1970‐1979	5274	4629	914	639
	1980‐1989	4985	4326	1121	760
	1990‐1993	1078	944	228	164
Year of entry to cohort	1972‐1979	3964	3560	100	77
	1980‐1989	5810	5000	1429	999
	1990‐1993	2764	2375	1409	1044
Country of residence	England and Wales	8078	7084	1255	910
	Scotland	4009	3395	1683	1210
	Northern Ireland	451	456		
Year of birth	<1930	162	151	712	612
	1930‐1949	890	702	1768	1222
	1950‐1969	5928	5072	458	286
	1970‐1993	5558	5010		
Total		12 538	10 935	2938	2120

^a^ Numbers in mortality analyses. The numbers in the cancer incidence analyses are similarly distributed but slightly smaller and with no subjects from Northern Ireland, total 27 682 (see Section 3).

During follow‐up for mortality, 8094 patients died, 1146 emigrated or otherwise left the NHS, 345 were censored when they reached age 85, and 18 946 reached the follow‐up end date alive and aged under 85. Follow‐up totalled 859 230 person‐years, a mean of 30.1 years per subject. The corresponding figures for cancer incidence follow‐up were all slightly less, with 27 682 patients followed, because follow‐up was not possible in Northern Ireland and the follow‐up period was slightly shorter (see Section 2). In total, 2234 cancers were recorded incident in the cohort during follow‐up and 8093 deaths, of which 876 were from cancer.

Overall cancer incidence risk in the cohort (Table 2) was significantly reduced for patients diagnosed with diabetes under age 30 (SIR = 0.89 [95% CI 0.84‐0.95]) and for those diagnosed at ages 30‐49 (0.89 [0.84‐0.95]). The only sites for which risk was significantly raised in the younger onset group were ovary (1.58 [1.16‐2.11]) and vulva (3.55 [1.94‐5.96]), while there were significantly reduced risks for cancers of the colon and rectum (0.79 [0.62‐0.98]), larynx (0.32 [0.07‐0.95]), lung (0.80 [0.63‐0.99]), prostate (0.51 [0.35‐0.71]) and kidney (0.62 [0.37‐0.97]) and Hodgkin lymphoma (0.43 [0.19‐0.81]). Risks were diminished but not significantly for cancers of the anus (0.44 [0.05‐1.59]) and cervix (0.73 [0.47‐1.07]), and not materially raised for cancer of the vagina (1.10 [0.03‐6.13]) (not in table). For patients with diabetes incident at ages 30‐49 there were significantly raised risks for cancers of the liver (1.76 [1.08‐2.72]) and kidney (1.46 [1.03‐2.00]), and significantly reduced risks for cancers of the lung (0.84 [0.71‐0.99]), non‐melanoma skin (0.71 [0.61‐0.83]), and prostate (0.61 [0.48‐0.78]).

**TABLE 2:** Cancer incidence risks in cohort by age at diagnosis of diabetes.

ICD9 code	Cancer site	Age at diabetes diagnosis (years)			
		0‐29		30‐49	
		No.	SIR (95% CI)	No.	SIR (95% CI)
141‐9	Tongue, mouth and pharynx	25	0.75 (0.48‐1.10)	17	0.77 (0.45‐1.23)
150	Oesophagus	21	1.03 (0.63‐1.57)	34	1.23 (0.86‐1.73)
151	Stomach	22	1.13 (0.71‐1.72)	31	1.03 (0.70‐1.46)
152	Small intestine	4	1.08 (0.30‐2.78)	2	0.70 (0.09‐2.54)
153‐4	Colon and rectum	77	0.79* (0.62‐0.98)	134	1.11 (0.93‐1.32)
155	Liver	5	0.45 (0.15‐1.06)	20	1.76* (1.08‐2.72)
156	Gallbladder	2	0.52 (0.06‐1.84)	7	1.44 (0.58‐2.96)
157	Pancreas	14	0.80 (0.44‐1.34)	28	1.20 (0.80‐1.74)
161	Larynx	3	0.32* (0.07‐0.95)	5	0.43 (0.14‐1.00)
162	Lung	81	0.80* (0.63‐0.99)	139	0.84* (0.71–0.99)
163	Pleura	4	1.07 (0.29‐2.73)	10	1.36 (0.65‐2.50)
170	Bone	5	0.89 (0.29‐2.08)	1	1.08 (0.03‐6.03)
171	Connective tissue	8	0.81 (0.35‐1.60)	3	0.79 (0.16‐2.31)
172	Melanoma	77	1.10 (0.87‐1.38)	21	0.88 (0.54‐1.34)
173	Non‐melanoma skin	254	0.93 (0.82‐1.05)	172	0.71*** (0.61–0.83)
174	Breast female	212	0.96 (0.83‐1.09)	87	0.82 (0.66‐1.02)
233.0	DCIS	27	1.20 (0.79‐1.75)	13	1.35 (0.72‐2.31)
180	Cervix	25	0.73 (0.47‐1.07)	10	1.55 (0.74‐2.84)
179, 182	Corpus uteri	18	0.85 (0.50‐1.34)	24	1.40 (0.91‐2.07)
183	Ovary	46	1.58** (1.16–2.11)	15	0.87 (0.49‐1.44)
184.0‐.4	Vulva	14	3.55*** (1.94‐5.96)	3	1.39 (0.29‐4.06)
185	Prostate	34	0.51*** (0.35‐0.71)	69	0.61*** (0.48–0.78)
186	Testis	28	0.75 (0.50‐1.09)	3	1.27 (0.26‐3.71)
188	Bladder	16	0.77 (0.44‐1.25)	37	0.98 (0.69‐1.35)
189	Kidney	19	0.62* (0.37‐0.97)	38	1.46* (1.03‐2.00)
190	Eye	3	0.99 (0.20‐2.90)	2	1.10 (0.13‐3.98)
191 192, 225 237.5, 237.6237.9239.6	Brain & nervous system, malignant and benign	45	0.93 (0.68‐1.25)	18	0.91 (0.54‐1.44)
193	Thyroid	13	0.66 (0.35‐1.12)	2	0.57 (0.07‐2.06)
196‐199	Cancer, unknown primary	11	0.47** (0.23‐0.84)	27	0.78 (0.51‐1.13)
200, 202	Non‐Hodgkin lymphoma	49	1.05 (0.78‐1.39)	34	1.09 (0.76‐1.53)
201	Hodgkin lymphoma	9	0.43** (0.19‐0.81)	4	1.43 (0.39‐3.67)
203	Myeloma	11	1.10 (0.55‐1.97)	6	0.49 (0.18‐1.08)
204‐8	Leukaemia	38	1.29 (0.91‐1.77)	22	1.08 (0.68‐1.63)
140‐172, 174‐208	All malignancies except NMSC	949	0.89*** (0.83‐0.94)	859	0.94 (0.88‐1.00)
140‐208	All malignancies	1203	0.89*** (0.84‐0.95)	1031	0.89*** (0.84‐0.95)

*Note*: *P < .05; **P < .01; ***P < .001.

Abbreviations: CI, confidence interval; DCIS, ductal carcinoma in situ of breast; ICD9, International Classification of Diseases 9th Revision; NMSC, non‐melanoma skin cancer; SIR, standardised incidence ratio.

Table 3 shows site‐specific cancer mortality in the cohort by age at diagnosis of diabetes. SMRs were close to unity for both ages at diagnosis, <30 (0.94 [0.84‐1.03]) and 30‐49 (0.97 [0.89‐1.06]). In the younger diagnosis age‐group there was a significantly raised mortality from non‐melanoma skin cancer (4.41 [1.43‐10.30]) and ovarian cancer (2.20 [1.42‐3.25]). In patients diagnosed at 30‐49 years, there were significantly raised risks of pancreatic cancer (1.49 [1.03‐2.08]) and renal cancer (1.89 [1.20‐2.83]), and a significantly decreased risk of lung cancer (0.79 [0.65‐0.95]).

**TABLE 3:** Cancer mortality risks in cohort by age at diagnosis of diabetes

ICD9 code	Cancer site	Age at diabetes diagnosis (years)			
		0‐29		30‐49	
		No.	SMR (95% CI)	No.	SMR (95% CI)
141‐9	Tongue, mouth and pharynx	7	0.69 (0.28‐1.43)	6	0.65 (0.24‐1.40)
150	Oesophagus	19	0.97 (0.58‐1.51)	31	1.19 (0.81‐1.68)
151	Stomach	10	0.76 (0.36‐1.39)	19	0.91 (0.54‐1.41)
152	Small intestine	1	0.78 (0.02‐4.34)	2	1.66 (0.20‐6.00)
153‐4	Colon and rectum	35	0.93 (0.65‐1.30)	52	1.00 (0.75‐1.31)
155	Liver	5	0.49 (0.16‐1.14)	12	1.10 (0.57‐1.92)
156	Gallbladder	2	1.24 (0.15‐4.49)	4	1.79 (0.49‐4.59)
157	Pancreas	14	0.79 (0.43‐1.32)	34	1.49* (1.03–2.08)
161	Larynx	1	0.36 (0.01‐2.00)	4	1.00 (0.27‐2.56)
162	Lung	79	0.91 (0.72‐1.13)	113	0.79* (0.65–0.95)
163	Pleura	2	1.18 (0.14‐4.25)	5	1.47 (0.48‐3.44)
170	Bone	4	1.34 (0.37‐3.44)	0	0.00 (0.00‐5.76)
171	Connective tissue	7	1.56 (0.63‐3.22)	1	0.49 (0.01‐2.71)
172	Melanoma	12	1.35 (0.70‐2.35)	1	0.19 (0.00‐1.07)
173	Non‐melanoma skin	5	4.41* (1.43–10.30)	2	1.42 (0.17‐5.13)
174	Breast female	36	0.85 (0.60‐1.18)	28	0.91 (0.61‐1.32)
180	Cervix	4	0.55 (0.15‐1.41)	2	0.63 (0.08‐2.28)
179, 182	Corpus uteri	3	0.84 (0.17‐2.45)	8	1.95 (0.84‐3.84)
183	Ovary	25	2.20** (1.42–3.25)	16	1.34 (0.77‐2.18)
184.0‐.4	Vulva	2	3.19 (0.39‐11.54)	2	3.02 (0.37‐10.89)
185	Prostate	5	0.45 (0.15‐1.04)	21	0.75 (0.47‐1.15)
186	Testis	0	0.00 (0.00‐2.56)	1	4.34 (0.11‐24.17)
188	Bladder	6	0.75 (0.27‐1.62)	8	0.54 (0.23‐1.06)
189	Kidney	6	0.57 (0.21‐1.25)	23	1.89** (1.20–2.83)
190	Eye	0	0.00 (0.00‐8.44)	1	2.56 (0.06‐14.27)
191, 192, 225, 237.5, 237.6, 237.9,239.6	Brain & nervous system, malignant and benign	25	1.08 (0.70‐1.59)	8	0.59 (0.26‐1.17)
193	Thyroid	1	1.21 (0.03‐6.77)	1	1.06 (0.03‐5.90)
196‐199	Cancer, unknown primary	17	0.68 (0.40‐1.10)	31	0.84 (0.57‐1.19)
200, 202	Non‐Hodgkin lymphoma	20	1.50 (0.92‐2.32)	15	1.15 (0.66‐1.88)
201	Hodgkin lymphoma	2	0.73 (0.09‐2.63)	2	1.98 (0.24‐7.16)
203	Myeloma	7	1.48 (0.60‐3.06)	6	0.80 (0.29‐1.74)
204‐8	Leukaemia	15	1.00 (0.56‐1.65)	15	1.31 (0.73‐2.17)
140‐172, 174‐208	All malignancies except NMSC	380	0.93 (0.84‐1.02)	489	0.97 (0.89‐1.06)
140‐208	All malignancies	385	0.94 (0.84‐1.03)	491	0.97 (0.89‐1.06)

*Note*: *P < .05; **P < .01; ***P < .001.

Abbreviations: CI, confidence interval; ICD9, International Classification of Diseases 9th Revision; NMSC, non‐melanoma skin cancer; SMR, standardised mortality ratio.

When we examined risks by duration since diabetes diagnosis for patients diagnosed with diabetes under age 30 (Table 4), there were no sites for which risk consistently increased with duration. Ovarian cancer risk was significantly raised at 10 to 19 years follow‐up (2.58 [1.11‐5.08]), and raised but not significantly thereafter; vulval cancer risk was significantly raised at 20‐29 (3.74 [1.02‐9.57]) and 30‐39 (5.23 [2.26‐10.30]) years of follow‐up; and prostate cancer risk was significantly raised at 10‐19 years (10.33 [1.25‐37.31]), but significantly decreased at 30‐39 (0.51 [0.25‐0.91]) and 40‐49 (0.47 [0.28‐0.73]) years. For patients whose diabetes was diagnosed at ages 30‐49, there was a consistent decrease in lung cancer risk with longer duration since diabetes diagnosis (*P* linear trend <.001), and a few significantly raised (nervous system) or diminished (non‐melanoma skin cancer, breast cancer, prostate cancer) risks in particular durations since diagnosis, with no obvious pattern.

**TABLE 4:** Cancer incidence risks in cohort by age at diagnosis of diabetes and duration since diagnosis, selected sites.

	Duration since diagnosis (years)									
	0‐		10‐		20‐		30‐		40‐	
Cancer site	No.	SIR (95% CI)	No.	SIR (95% CI)	No.	SIR (95% CI)	No.	SIR (95% CI)	No.	SIR (95% CI)
Diagnosis age < 30 years										
Oesophagus	0	0.0 (0.00‐72.64)	1	1.82 (0.05‐10.12)	2	0.63 (0.08‐2.27)	9	1.21 (0.55‐2.29)	9	0.98 (0.45‐1.86)
Stomach	0	0.0 (0.00‐25.35)	0	0.00 (0.00‐3.71)	8	2.25 (0.97‐4.43)	7	1.07 (0.43‐2.21)	7	0.87 (0.35‐1.79)
Colon and rectum	2	3.56 (0.43‐12.84)	1	0.26 (0.01‐1.44)	25	1.44 (0.93‐2.13)	17	0.48** (0.28‐0.77)	32	0.79 (0.54‐1.12)
Pancreas	0	0.00 (0.00‐49.66)	1	1.89 (0.05‐10.50)	2	0.75 (0.09‐2.71)	7	1.11 (0.45‐2.29)	4	0.50 (0.14‐1.29)
Lung	0	0.00 (0.00‐14.00)	1	0.44 (0.01‐2.44)	15	1.15 (0.64‐1.89)	23	0.68 (0.43‐1.02)	42	0.82 (0.59‐1.10)
Melanoma	2	0.99 (0.12‐3.58)	9	0.90 (0.41‐1.70)	30	1.35 (0;91‐1.92)	26	1.08 (0.70‐1.58)	10	0.88 (0.42‐1.62)
Non‐melanoma skin cancer	1	0.56 (0.01‐3.13)	12	0.90 (0.46‐1.57)	48	0.86 (0.63‐1.14)	94	0.90 (0.73‐1.10)	99	1.02 (0.83‐1.24)
Breast female	0	0.00 (0.00‐2.99)	16	1.17 (0.67‐1.91)	63	1.05 (0.81‐1.35)	89	0.92 (0.74‐1.14)	44	0.88 (0.64‐1.18)
Ovary	0	0.00 (0.00‐5.06)	8	2.58* (1.11–5.08)	11	1.39 (0.69‐2.49)	15	1.42 (0.79‐2.34)	12	1.78 (0.92‐3.11)
Vulva	0	0.00 (0.00‐94.44)	2	6.91 (0.84‐24.96)	4	3.74* (1.02‐9.57)	8	5.23*** (2.26‐10.30)	0	0.00 (0.00‐3.70)
Prostate	0	0.00 (0.00‐120.04)	2	10.33** (1.25‐37.31)	2	0.47 (0.06‐1.69)	11	0.51* (0.25‐0.91)	19	0.47*** (0.28‐0.73)
Bladder	1	4.07 (0.10‐22.67)	2	1.99 (0.24‐7.18)	4	1.32 (0.36‐3.38)	4	0.61 (0.17‐1.57)	5	0.50 (0.16‐1.17)
Kidney	0	0.00 (0.00‐8.98)	0	0.00 (0.00‐2.90)	5	0.82 (0.27‐1.91)	11	0.89 (0.44‐1.59)	3	0.29* (0.06‐0.84)
Brain and nervous system, malignant and benign	3	0.71 (0.15‐2.06)	3	0.41 (0.08‐1.19)	19	1.38 (0.83‐2.16)	13	0.87 (0.47‐1.49)	7	0.89 (0.36‐1.82)
Non‐Hodgkin lymphoma	2	0.92 (0.11‐3.34)	4	0.72 (0.20‐1.84)	16	1.35 (0.77‐2.20)	15	0.97 (0.54‐1.61)	12	1.05 (0.54‐1.83)
Hodgkin lymphoma	4	1.13 (0.31‐2.91)	1	0.14* (0.00‐0.80)	2	0.33 (0.04‐1.18)	2	0.58 (0.07‐2.08)	0	0.00 (0.00‐3.44)
Leukaemia	2	0.47 (0.06‐1.70)	2	0.46 (0.06‐1.65)	8	1.29 (0.56‐2.54)	14	1.79 (0.98‐3.01)	12	1.76 (0.91‐3.08)
All malignancies except NMSC	30	1.05 (0.71‐1.49)	73	0.79* (0.62‐0.99)	265	1.08 (0.96‐1.22)	325	0.86** (0.77‐0.96)	256	0.78*** (0.69‐0.88)
All malignancies	31	1.02 (0.69‐1.44)	85	0.80* (0.64‐0.99)	313	1.04 (0.93‐1.16)	419	0.87** (0.79‐0.96)	355	0.83** (0.75‐0.93)

	Duration since diagnosis (years)									
	0‐		10‐		20‐		30‐		40‐	
Cancer site	No.	SIR (95% CI)	No.	SIR (95% CI)	No.	SIR (95% CI)	No.	SIR (95% CI)	No.	SIR (95% CI)
Diagnosis age 30‐49 years										
Oesophagus	0	0.00 (0.00‐4.35)	3	0.59 (0.12‐1.72)	17	1.57 (0.91‐2.51)	9	1.09 (0.50‐2.07)	5	2.22 (0.72‐5.17)
Stomach	0	0.00 (0.00‐3.34)	7	1.22 (0.49‐2.51)	12	1.04 (0.54‐1.82)	8	0.89 (0.38‐1.75)	4	1.57 (0.43‐4.03)
Colon and rectum	3	0.79 (0.16‐2.32)	26	1.21 (0.79‐1.78)	54	1.15 (0.87‐1.51)	37	1.01 (0.71‐1.39)	14	1.33 (0.73‐2.23)
Pancreas	0	0.00 (0.00‐5.30)	6	1.47 (0.54‐3.21)	11	1.23 (0.61‐2.19)	8	1.11 (0.48‐2.18)	3	1.42 (0.29‐4.15)
Lung	7	1.46 (0.59‐3.01)	33	1.10 (0.76‐1.55)	58	0.88 (0.67‐1.14)	36	0.71* (0.50‐0.99)	3	0.22** (0.05‐0.65)
Melanoma	1	0.60 (0.02‐3.32)	4	0.76 (0.21‐1.95)	10	1.13 (0.54‐2.07)	6	0.97 (0.36‐2.11)	0	0.00 (0.00‐2.13)
Non‐melanoma skin cancer	8	1.21 (0.52‐2.39)	30	0.83 (0.56‐1.19)	48	0.54*** (0.40‐0.72)	68	0.84 (0.65‐1.07)	18	0.68 (0.40‐1.07)
Breast female	9	1.00 (0.46‐1.89)	28	0.92 (0.61‐1.33)	26	0.66* (0.43‐0.96)	15	0.70 (0.39‐1.15)	9	1.84 (0.84‐3.49)
Ovary	1	0.79 (0.02‐4.40)	6	1.29 (0.47‐2.81)	5	0.76 (0.25‐1.78)	2	0.53 (0.06‐1.90)	1	1.13 (0.03‐6.27)
Vulva	1	10.38 (0.26‐57.83)	0	0.00 (0.00‐9.03)	0	0.00 (0.00‐4.57)	2	3.10 (0.38‐11.19)	0	0.00 (0.00‐19.02)
Prostate	0	0.00 (0.00‐6.04)	8	0.65 (0.28‐1.29)	26	0.56** (0.36‐0.82)	26	0.63* (0.41‐0.93)	9	0.84 (0.38‐1.59)
Bladder	1	0.70 (0.02‐3.92)	5	0.71 (0.23‐1.65)	17	1.20 (0.70‐1.92)	7	0.61 (0.25‐1.26)	7	2.09 (0.84‐4.31)
Kidney	2	2.03 (0.25‐7.33)	7	1.40 (0.56‐2.89)	15	1.45 (0.81‐2.39)	12	1.60 (0.82‐2.79)	2	0.98 (0.12‐3.54)
Brain & nervous system, malignant and benign	1	0.72 (0.02‐4.03)	2	0.42 (0.05‐1.52)	5	0.67 (0.22‐1.55)	10	2.10* (1.01‐3.86)	0	0.00 (0.00‐3.12)
Non‐Hodgkin lymphoma	1	0.60 (0.02‐3.34)	9	1.39 (0.64‐2.64)	11	0.93 (0.46‐1.66)	11	1.28 (0.64‐2.29)	2	0.86 (0.10‐3.10)
Hodgkin lymphoma	0	0.00 (0.00‐9.72)	3	3.94 (0.81‐11.51)	1	1.05 (0.03‐5.85)	0	0.00 (0.00‐6.82)	0	0.00 (0.00‐26.90)
Leukaemia	2	2.44 (0.30‐8.80)	5	1.32 (0.43‐3.08)	11	1.42 (0.71‐2.54)	3	0.49 (0.10‐1.43)	1	0.57 (0.01‐3.16)
All malignancies except NMSC	42	1.08 (0.78‐1.46)	195	1.09 (0.94‐1.25)	333	0.93 (0.84‐1.04)	219	0.83** (0.72‐0.95)	68	0.96 (0.75‐1.22)
All malignancies	50	1.10 (0.82‐1.45)	225	1.04 (0.91‐1.19)	381	0.86** (0.77‐0.95)	287	0.83** (0.74‐0.94)	86	0.88 (0.71‐1.09)

*Note*: *P < .05; **P < .01; ***P < .001.

Abbreviations: CI, confidence interval; NMSC, non‐melanoma skin cancer; SIR, standardised incidence ratio.

Analysis of incidence risks in the first year after diabetes diagnosis (not in table), gave no evidence of diagnostic bias as a reason for raised risks in the follow up overall: for patients diagnosed with diabetes under age 30, there were only four cancers diagnosed in the first year—two melanomas, one bone cancer and one nervous system cancer; the risk for all malignancies other than non‐melanoma skin cancer was 2.63 (0.72‐6.75). In patients diagnosed with diabetes at ages 30‐49 there were no cancers diagnosed in the first year after diabetes diagnosis. Analyses of cancer mortality risks by duration since diabetes diagnosis (not in table) showed nothing beyond the incidence analyses, and again a consistent trend of diminishing lung cancer risks with longer follow up for patients with diabetes diagnosed at ages 30‐49.

When we subdivided cancer incidence risk more finely by age at diagnosis of diabetes in 10‐year strata (Table 5), for most cancer sites there was no obvious relation beyond the differences noted above between those aged under 30 and 30‐49 at diagnosis. However, for ovarian cancer there was a much greater (and significant) risk for those diagnosed at ages 10‐19 (RR 1.91 [1.24‐2.82]) than at other ages; for vulval cancer far greater RRs for diagnosis at ages 0‐9 (5.10 [1.66‐11.91]) and 10‐19 (4.89 [2.24‐9.29]) than at older ages. For Hodgkin lymphoma there was a highly significant reduced risk for those diagnosed under age 10 (0.11 [0.00‐0.63]), whereas diminutions in risk were far less, and not significant, for diagnoses at older ages. Conversely, liver cancer risk was only significantly raised for diabetics diagnosed at ages 40‐49 (2.30 [1.23‐3.94]) and non‐melanoma skin cancer risk only significantly diminished for patients diagnosed at ages 20‐29 (0.77 [0.60‐0.96]), 30‐39 (0.73 [0.58‐0.90]) and 40‐49 (0.70 [0.56‐0.86]). Subdividing further the group of females aged 10‐19 at diagnosis (see Section 2; not in table), there was a marked tendency for risks of ovarian and vulval cancers to be particularly great for those diagnosed at ages 10‐14 (2.06 (1.26‐3.18) n = 20 and 5.94 (2.56‐11.70) n = 8, respectively).

**TABLE 5:** Cancer incidence risks in cohort by age at diagnosis of diabetes: selected sites.

Cancer site	Age at diabetes diagnosis (years)									
	0‐9		10‐19		20‐29		30‐39		40‐49	
	No.	SIR (95% CI)	No.	SIR (95% CI)	No.	SIR (95% CI)	No.	SIR (95% CI)	No.	SIR (95% CI)
Tongue, mouth & pharynx	6	0.90 (0.33‐1.97)	14	0.90 (0.49‐1.51)	5	0.44 (0.14‐1.03)	5	0.41* (0.13‐0.96)	12	1.21 (0.63‐2.12)
Oesophagus	2	0.80 (0.10‐2.89)	9	1.11 (0.51‐2.10)	10	1.02 (0.49‐1.87)	18	1.36 (0.80‐2.15)	16	1.12 (0.64‐1.82)
Stomach	3	1.06 (0.22‐3.11)	12	1.60 (0.83‐2.80)	7	0.77 (0.31‐1.59)	13	0.98 (0.52‐1.68)	18	1.06 (0.63‐1.67)
Colon and rectum	11	0.70 (0.35‐1.25)	34	0.86 (0.59‐1.20)	32	0.75 (0.52‐1.07)	63	1.12 (0.86‐1.43)	71	1.11 (0.87‐1.40)
Liver	2	1.09 (0.13‐3.95)	3	0.64 (0.13‐1.88)	0	0.00* (0.00‐0.81)	7	1.23 (0.49‐2.53)	13	2.30* (1.23–3.94)
Pancreas	1	0.40 (0.01‐2.25)	4	0.57 (0.16‐1.46)	9	1.12 (0.51‐2.12)	14	1.30 (0.71‐2.18)	14	1.12 (0.61‐1.88)
Larynx	0	0.00 (0.00‐3.17)	3	0.82 (0.17‐2.38)	0	0.00* (0.00‐0.83)	3	0.51 (0.11‐1.49)	2	0.34 (0.04‐1.25)
Lung	12	1.07 (0.55‐1.86)	19	0.52** (0.31‐0.82)	50	0.93 (0.69‐1.23)	66	0.87 (0.68‐1.11)	73	0.81 (0.64‐1.02)
Melanoma	26	1.17 (0.77‐1.72)	36	1.07 (0.75‐1.49)	15	1.05 (0.59‐1.73)	15	1.15 (0.64–1.89)	6	0.56 (0.20‐1.21)
Non‐melanoma skin	55	0.98 (0.74‐1.28)	125	1.04 (0.86‐1.24)	74	0.77* (0.60–0.96)	86	0.73** (0.58–0.90)	86	0.70*** (0.56–0.86)
Breast female	57	1.05 (0.79‐1.36)	95	0.89 (0.72‐1.09)	60	0.99 (0.76‐1.27)	44	0.79 (0.58‐1.07)	43	0.85 (0.62‐1.15)
DCIS	7	1.35 (0.54‐2.79)	8	0.72 (0.31‐1.42)	12	1.95* (1.01‐3.40)	9	1.64 (0.75‐3.11)	4	0.97 (0.26‐2.48)
Cervix	10	0.78 (0.37‐1.43)	15	0.92 (0.51‐1.51)	0	0.00* (0.00‐0.72)	4	1.13 (0.31‐2.88)	6	2.06 (0.76‐4.48)
Corpus uteri	1	0.27 (0.01‐1.49)	6	0.67 (0.24‐1.45)	11	1.29 (0.65‐2.32)	11	1.24 (0.62‐2.22)	14	1.57 (0.86‐2.63)
Ovary	10	1.29 (0.62‐2.37)	25	1.91** (1.24–2.82)	11	1.33 (0.66‐2.38)	7	0.84 (0.34‐1.74)	8	0.90 (0.39‐1.77)
Vulva	5	5.10** (1.66–11.91)	9	4.89*** (2.24–9.29)	0	0.00 (0.00‐3.30)	0	0.00 (0.00‐3.57)	3	2.67 (0.55‐7.80)
Prostate	6	1.03 (0.38‐2.25)	11	0.43** (0.22‐0.77)	17	0.48** (0.28‐0.76)	36	0.67* (0.47‐0.92)	33	0.56*** (0.39‐0.79)
Testis	8	0.54 (0.23‐1.07)	18	0.98 (0.58‐1.54)	2	0.51 (0.06‐1.83)	2	1.14 (0.14‐4.13)	1	1.63 (0.04‐9.07)
Bladder	4	1.61 (0.44‐4.11)	7	0.91 (0.37‐1.88)	5	0.47 (0.15‐1.09)	19	1.15 (0.69‐1.80)	18	0.84 (0.50‐1.33)
Kidney	3	0.50 (0.10‐1.45)	9	0.66 (0.30‐1.26)	7	0.63 (0.26‐1.31)	20	1.52 (0.93‐2.35)	18	1.39 (0.83‐2.20)
Brain & nervous system, malignant and benign	14	0.89 (0.48‐1.49)	21	0.95 (0.59‐1.45)	10	0.98 (0.47‐1.81)	11	1.07 (0.53‐1.92)	7	0.74 (0.30‐1.53)
Thyroid	7	0.91 (0.37–1.88)	4	0.42 (0.12‐1.08)	2	0.74 (0.09‐2.66)	2	1.00 (0.12‐3.61)	0	0.00 (0.00‐2.44)
Cancer, Unknown Primary	1	0.27 (0.01‐1.50)	4	0.44 (0.12‐1.12)	6	0.57 (0.21‐1.24)	12	0.80 (0.41‐1.39)	15	0.77 (0.43‐1.26)
Non‐Hodgkin lymphoma	19	1.60 (0.96‐2.50)	15	0.71 (0.40‐1.17)	15	1.11 (0.62‐1.83)	15	0.96 (0.54‐1.59)	19	1.22 (0.73‐1.90)
Hodgkin lymphoma	1	0.11** (0.00–0.63)	7	0.69 (0.28‐1.42)	1	0.47 (0.01‐2.62)	1	0.64 (0.02‐3.56)	3	2.45 (0.51‐7.17)
Myeloma	0	0.00 (0.00‐2.40)	6	1.44 (0.53‐3.14)	5	1.16 (0.38‐2.71)	2	0.35 (0.04‐1.27)	4	0.62 (0.17‐1.59)
Leukaemia	11	1.18 (0.59‐2.11)	16	1.28 (0.73‐2.08)	11	1.43 (0.72‐2.56)	8	0.83 (0.36‐1.63)	14	1.31 (0.71‐2.19)
All malignancies except NMSC	234	0.97 (0.85‐1.11)	418	0.89* (0.81‐0.98)	297	0.82*** (0.73‐0.92)	407	0.92 (0.83‐1.02)	452	0.95 (0.87‐1.04)
All malignancies	289	0.97 (0.87‐1.09)	543	0.92 (0.85‐1.00)	371	0.81*** (0.73‐0.89)	493	0.88** (0.81‐0.96)	538	0.90* (0.83‐0.98)

*Note*: *P < .05; **P < .01; ***P < .001.

Abbreviations: CI, confidence interval; DCIS, ductal carcinoma in situ of breast; SIR, standardised incidence ratio.

In analyses dividing age at diagnosis into 0‐6, 7‐12 and 13‐29 (see Section 2; not in table), there were no findings appreciably beyond those above: relative risks for ovarian cancer and vulval cancer were greatest for patients diagnosed with diabetes at ages 7‐12 (2.09 [1.31‐3.17] and 5.61 [2.42‐11.05], respectively), similar to those above for diagnosis at ages 10‐14.

In analyses of cancer mortality by age at diabetes incidence (not in table), the greatest risks for ovarian and vulval cancers were at ages 10‐19 (3.47 [1.98‐5.64] and 7.91 [0.96‐28.59], respectively), but there were no obvious trends although a notably high risk of death from non‐melanoma skin cancer in patients diagnosed with diabetes at ages 10‐19 (8.46 [2.31‐21.67]).

Analyses of incidence risks by sex (not in table) showed that the reduced risk of cancer overall arose entirely from males: relative risks in males were 0.78 (0.71‐0.85; *P* < 0.001) for those with diabetes incident under age 30, and 0.83 (0.77‐0.90; *P* < 0.001) for those diagnosed at ages 30‐49, whereas for females the RRs were 1.00 (0.93‐1.08) and 0.99 (0.90‐1.09), respectively. The male/female difference was particularly marked for gastrointestinal tract cancers in patients diagnosed with diabetes under age 30:0.68 (0.53‐0.85; *P* < .001) for males and 1.10 (0.86‐1.39) for females, for gastrointestinal cancers in total excluding peritoneum. For cancer mortality (not in table) the pattern was similar.

## 4 DISCUSSION

Our data on a large cohort of insulin‐treated patients with diabetes, now followed for an average of 30 years and many for over 40 years, showed different patterns of cancer risk for those diagnosed with diabetes under age 30, almost all with type 1 diabetes^18^ and who form the focus of this paper, and those diagnosed at ages 30 to 49, who are likely to have been predominantly type 2,^20^ and whom we include as a comparison from the same cohort. For the former, there were significantly raised risks of only two cancer sites, ovary and vulva, and there were significantly decreased risks of cancers of the colon and rectum, larynx, lung, prostate and kidney and of Hodgkin lymphoma. Patients diagnosed with diabetes at ages 30 to 49 also had significantly reduced risks of cancers of the lung and prostate as well as non‐melanoma skin cancer, but significantly raised risks only of liver and kidney cancers. There were, however, also somewhat raised risks, albeit not significantly, in this older age group but not the younger, for several cancers that have been shown increased in patients with diabetes generally (ie, preponderantly type 2)^1^, ^2^, ^3^, ^4^, ^5^—liver, pancreas, endometrium, colorectum and non‐Hodgkin lymphoma.

Previous studies of cancer risks in patients with type 1 diabetes^5^, ^6^, ^7^, ^8^, ^9^, ^10^, ^11^, ^12^, ^13^, ^25^, ^26^ have produced very inconsistent results,^27^ particularly in the extent to which risks were raised for cancer sites that are at high risk in patients with diabetes overall (ie, effectively, in type 2 patients). However, these studies have varied greatly in the parameters used to define diabetes type and in the length of follow up after diabetes diagnosis, and these variations may explain some or all of the inconsistency between results and the differences from our findings. Most of the studies have been relatively small, but studies from Sweden^9^, ^10^, ^13^ and Australia^5^ have been large, as has an analysis combining heterogeneous data from these countries plus Finland, Denmark and Scotland,^12^ the latter likely with a modest overlap with the Scottish patients within our whole UK study.

In our cohort, unlike several^8^, ^9^, ^10^, ^12^, ^13^ but not all^5^, ^6^, ^7^, ^12^, ^25^ others, all patients were treated with insulin. While this does not ensure that they were type 1, insulin treatment is a prerequisite for type 1 diagnosis so cohorts that included non‐insulin treated patients will, at least to that extent, have included non‐type 1 patients. At the time of our recruitment, almost all insulin‐treated patients with younger ages at diabetes diagnosis were type 1^20^: we estimate that at least 94% of subjects in our cohort diagnosed at ages under 30 were type 1,^18^ whereas for the 30 to 49 onset group we estimate that most were type 2 (68% based on data published by Laakso and Pyorala^20^). In studies not restricted to insulin‐treated patients, and/or with inclusion of patients diagnosed up to age 40^12^, ^13^ or 45^5^ or older,^6^, ^8^ the proportion who were type 2 will have been considerable. This is the more so if the subjects were from more recent diagnosis periods: the great majority of patients with diabetes incident at ages ≥30 are type 2^14^, ^15^, ^16^ and the proportion who are type 2 has been increasing over time.^15^, ^28^, ^29^ Furthermore, since the subjects who enter follow‐up older, and hence are more likely to be type 2, will have had more of their follow‐up at ages at which cancer incidence rates are greatest, they will disproportionately contribute to cancer incidence in a cohort. For instance, although only 8.5% of our cohort were aged 40 to 49 at diabetes diagnosis, this age group contributed 24.1% of the cancers incident (Table 4).

Differences between our results and those previously may therefore reflect differences in the extent to which nominally type 1 cohorts inadvertently included type 2 patients (assuming that cancer risks truly differ between these two diabetes types, which seems likely if only because the latter is strongly associated with a major known cancer risk factor [obesity] whereas the former is not).

The two cancers at significantly raised risk in our young‐onset diabetes cohort, were both of the female reproductive tract—ovarian and vulval cancers. Most previous cohort studies have found raised ovarian cancer risks in nominally type 1 patients,^5^, ^10^, ^11^, ^12^ and the exceptions were small with wide confidence intervals.^8^, ^9^ While the risk has not previously been examined by age at diabetes diagnosis, we found greatest risk for women whose diabetes was diagnosed at ages 10 to 14, the age of menarche for 90% of women in the UK.^21^ However, these are subgroup analyses which need to be interpreted cautiously. Although we do not have data directly on age at menarche in our cohort, the results imply that the raised ovarian cancer risk might be due to greater susceptibility of the ovary around puberty to the metabolic disruption accompanying the initial disturbance leading to diabetes diagnosis, in the same way as breast cancer risk is more raised after radiation exposure at puberty than at other times.^30^ Vulval cancer too showed greatest (6‐fold) risk for those whose diabetes had been diagnosed at ages 10 to 14, although also with greatly raised risk for those diagnosed at younger ages than this (0‐9 years); the reasons are inapparent, but for both tumours an analysis of risk in relation to actual age at menarche, if there were a cohort in which this was ascertained, would be desirable. We can find no previous cohort analyses of vulval cancer risk in patients with type 1 diabetes. Two cohorts have examined risk of a wider category, “other female genital cancers”, with non‐significant results, but based on only four and three cases, respectively.^8^, ^10^ One cohort examined risk of non‐cervical anogenital tumours overall, with non‐significantly raised risk for cancers and significantly raised risk for intraepithelial neoplasms.^31^ No cohorts have published data on ovarian or vulval cancer risks by age at diabetes onset. Vulval cancer at younger ages is often HPV related,^31^, ^32^ as are anal, vaginal and cervical cancers, but there was no raised risk of these other tumours in our cohort. An alternative potential mechanism is that glycosuria may cause chronic itching, inflammation, and lichen sclerosis, which are posited as potential risk factors for vulval cancer.^32^, ^33^

Of the other significant findings in the patients with diabetes diagnosed under age 30, the diminished risks for larynx and lung cancers presumably reflect diminished smoking, which has been found in some but not all studies of young people with type 1 diabetes.^34^, ^35^ This accords also with the diminished risks, although not significant, in our cohort for other smoking and alcohol‐related cancers—oropharyngeal, pancreatic, liver and bladder, implying a healthy lifestyle at least in regard to these factors. However, a contribution to diminished risk could also have occurred if the combined effects of smoking and diabetes on mortality at earlier ages had led to selectively high mortality from non‐cancer causes (notably cardiovascular) in smokers in the cohort compared with smokers in the general population, leaving fewer smokers to develop cancer during prolonged follow‐up.

The diminished risk of prostate cancer that we found has been seen also in other large nominally type 1 diabetes cohorts^5^, ^12^ although not in a cohort in Taiwan.^11^ It might be a consequence of reduced free testosterone levels.^36^, ^37^ There was also a significantly diminished risk of Hodgkin lymphoma, primarily among patients whose diabetes onset had been before age 10. Previous studies of nominally type 1 cohorts have found non‐significantly reduced risks of Hodgkin lymphoma^5^, ^10^ or risk decreased in females but not males,^12^ but none have examined this by age at diabetes onset. Epidemiological studies have suggested that the aetiology of Hodgkin lymphoma may rest in lack of childhood exposure to an infectious agent that is harmless if encountered young, but can cause lymphoma if caught later as a consequence of relative social isolation in childhood.^38^ However, one might expect that childhood diabetes would if anything lead to less, not more, mixing with peers and hence to higher Hodgkin lymphoma risk, not lower (unless childhood hospitalisation led to the relevant infection).

The cancer risks in our cohort for patients with diabetes diagnosed at ages 30 to 49, likely to be mainly type 2 cases, were much more similar to those in previous cohorts. The significantly raised risk of liver and kidney cancers, and non‐significantly raised risks of colorectal, pancreatic and endometrial cancers and non‐Hodgkin lymphoma, accord with raised risks of these tumours generally found in type 2 cohorts^1^, ^2^, ^3^, ^4^, ^5^ and, with the exception of non‐Hodgkin lymphoma, generally also in nominally type 1 cohorts.^5^, ^8^, ^9^, ^10^, ^11^, ^12^

Likewise, the significantly reduced risk of prostate cancer in patients with diabetes diagnosed at older ages in our cohort accords with reduced risk in type 2 diabetes cohorts^1^, ^2^, ^3^, ^4^, ^5^ and might, as noted above, relate to testosterone levels. The significantly reduced risk of lung cancer in our older onset diabetes patients, as in the younger onset group, seems likely to be due to reduced smoking. There were not reduced risks of lung cancer in other large type 1 cohorts, but perhaps smoking patterns in those cohorts may have been different.

Our results for cancer mortality showed similar patterns to those for cancer incidence, although less marked based on smaller numbers, and with a modest tendency to greater SMRs than SIRs, perhaps indicating poorer survival from cancer in the patients with diabetes than in the general population. The only previous cohort study of site‐specific cancer mortality in patients with type 1 diabetes^5^ likewise found somewhat greater SMRs than SIRs.

The results on risk by duration since diabetes onset in our cohort gave no indication of relations other than for ovarian and vulval cancer risks in patients with diabetes onset before age 30, which reached a peak and then diminished, and lung cancer in patients with diabetes onset at ages 30 and older. The latter diminished consistently with longer follow up, and may indicate selective early mortality of heavy smokers with diabetes (which we estimate based on published data on mortality^39^ and smoking prevalence^40^ in patients with diabetes might have reduced the proportion of heavy smokers in the denominator by about 40%), and/or younger smoking cessation in patients with diabetes, and hence exceptionally low smoking levels in those surviving to later years. With the exception of two cases of melanoma, there was no indication of raised cancer risk soon after the start of follow up, which could indicate cancer diagnoses as a consequence of clinical workup for diabetes or vice versa. There were no cases of pancreatic cancer in the first few years after diabetes onset, which if present could have indicated reverse causation bias. There appear to have been no previous published studies of cancer risks by duration since diabetes onset in type 1 patients. Analyses of risks by duration since hospitalisation or since registration in type 1 cohorts^9^, ^10^, ^12^ have shown greatly raised risks of several cancer sites in the first year after entry and no marked pattern thereafter; however, the first year risks might have been an artefact of entry at hospitalisation, which might have been occasioned by cancer rather than diabetes.

Our study of type 1 diabetes patients had several strengths—large size; information to allow analyses by age at diagnosis of diabetes (not published for any other cohort, except for one publication on a two‐way split of ages for three cancer sites^10^); high completeness of follow‐up; longer follow‐up than any study previously; information on cancer mortality as well as incidence; and restriction to insulin‐treated patients. However, it is a weakness that, like most other cohorts, we did not have direct data on diabetes type: for the diabetes onset under age 30 group, the effect should have been minor, since we estimate about 94% will have been type 1, but for the comparison group of patients diagnosed at ages 30 to 49, the effect will have been of greater impact since only about 68% would have been type 2. We used standard quinquennial and decennial cut‐points for age and duration (plus immunologically relevant cut‐points reported by others,^22^ and a 1‐year cut‐point examining for potential diagnostic artefact at the time of diagnosis) to avoid data‐driven analyses. However, since these are subgroup analyses, they need to be interpreted cautiously, especially where numbers are sparse.

In summary, our large cohort of patients with type 1 diabetes has shown significantly raised risks of ovarian and vulval cancers, greatest in patients diagnosed with diabetes at ages 10 to 14, the usual ages at puberty in the UK, and an overall diminished risk of cancer, reflecting diminished risks of smoking and alcohol‐related cancers, likely because of healthier lifestyles in these respects of the diabetes patients, and of prostate cancer, which might be connected to the reduced testosterone levels in men with diabetes.

## AUTHOR CONTRIBUTIONS

Anthony J. Swerdlow, Stefan D. Slater, Andrew C. F. Burden, Johannes L. Botha, Norman R. Waugh, Wendy Gatling and Christopher C. Patterson contributed to, the original assembly of the BDA cohort. Stefan D. Slater, Anthony J. Swerdlow, Andrew C. F. Burden, Johannes L. Botha, Norman R. Waugh, Andrew D. Morris, Wendy Gatling, Kathleen M. Gillespie and Christopher C. Patterson are or were responsible for the assembly and/or continuation of the component diabetes registers. Anthony J. Swerdlow, Christopher C. Patterson and Minouk J. Schoemaker conducted the continuing follow up. Minuok J. Schoemaker and Michael E. Jones conducted the data handling and analyses. Anthony J. Swerdlow drafted the manuscript. All authors contributed to and signed off on the final manuscript. The work reported in the paper has been performed by the authors, unless clearly specified in the text.

## FUNDING INFORMATION

We thank the British Diabetic Association and Medical Research Council for funding of the cohort at its outset.

## CONFLICT OF INTEREST

Minock J. Schoemaker is employed by IQVIA, a contract research organisation. IQVIA did not sponsor this work. The other authors declare no conflict of interest.

## ETHICS STATEMENT

This register‐based study was approved by the BMA central ethics committee for conduct without approaches to individuals.

## Data Availability

The data that support the findings of this study are available from the corresponding author upon reasonable request.
